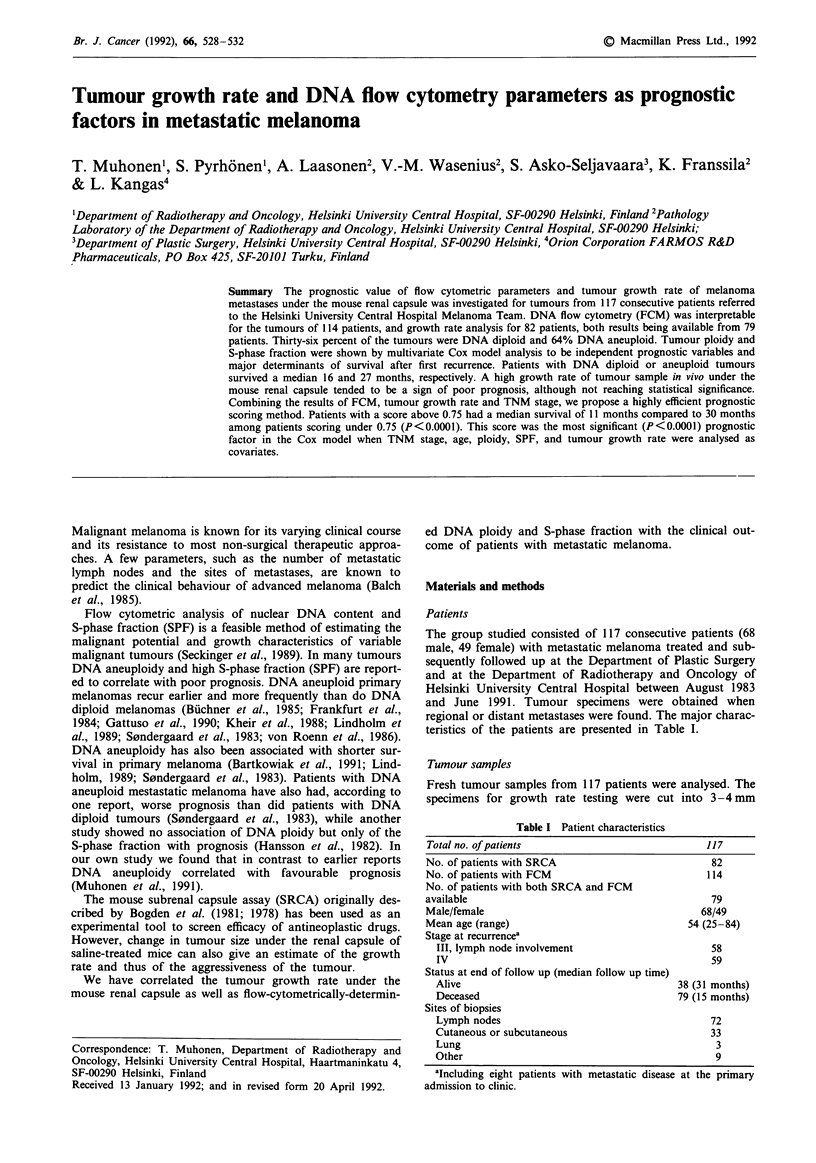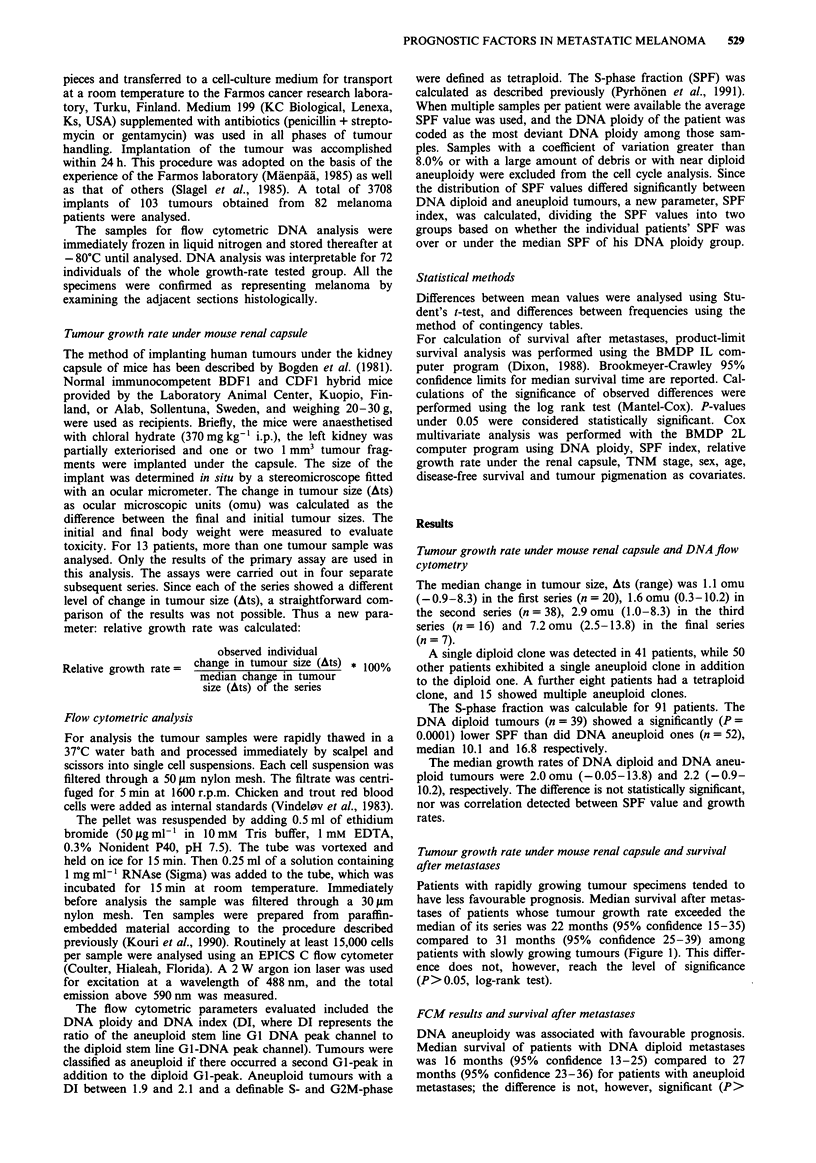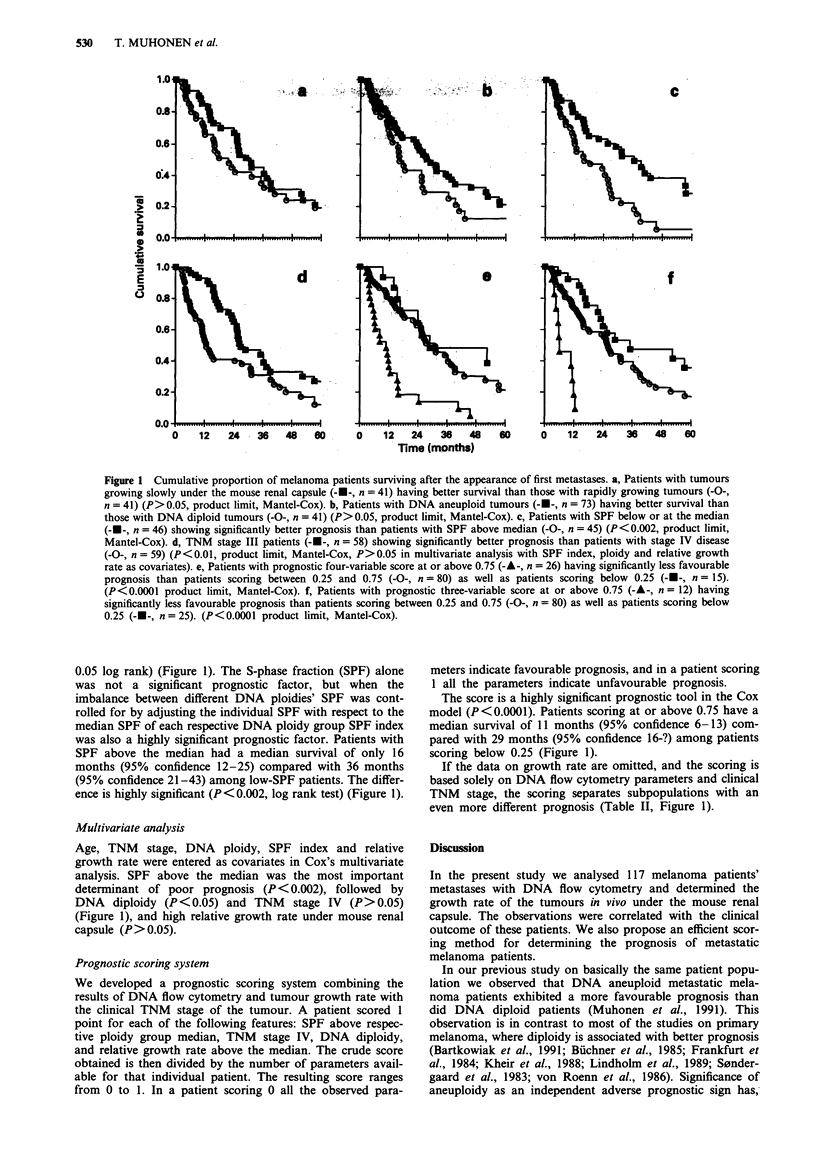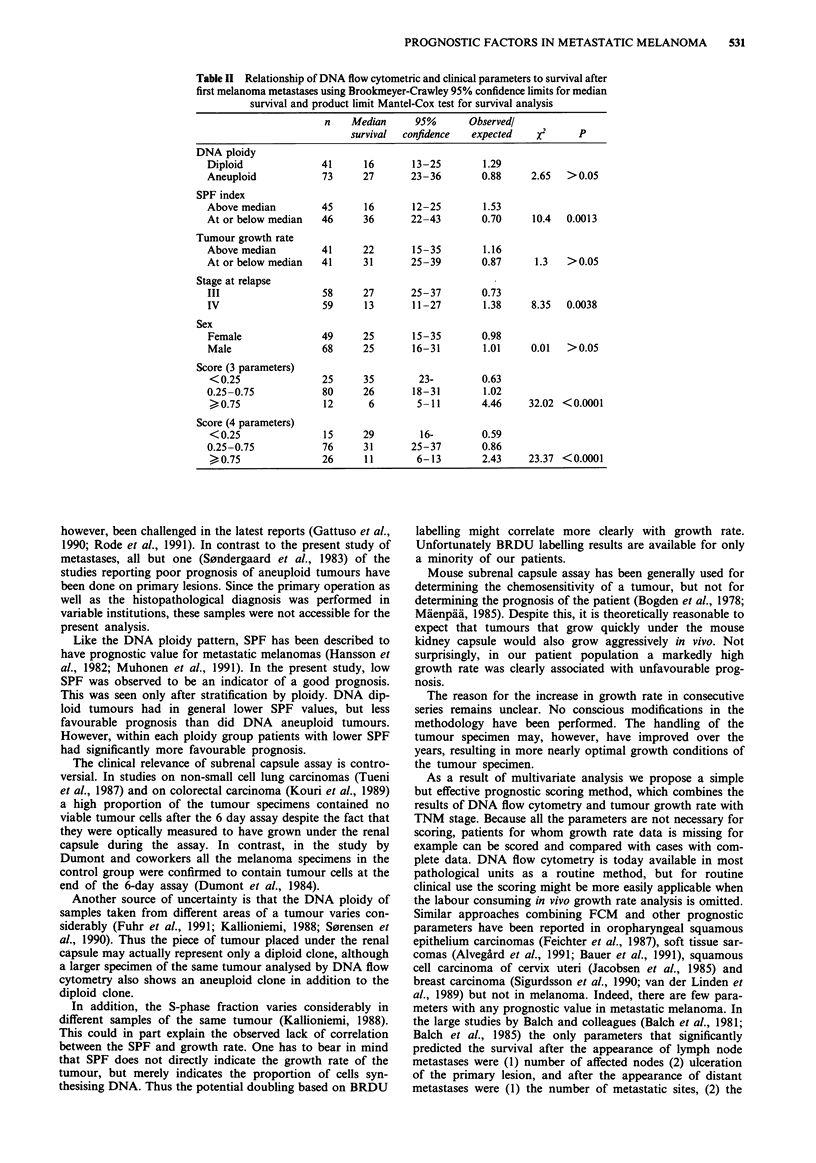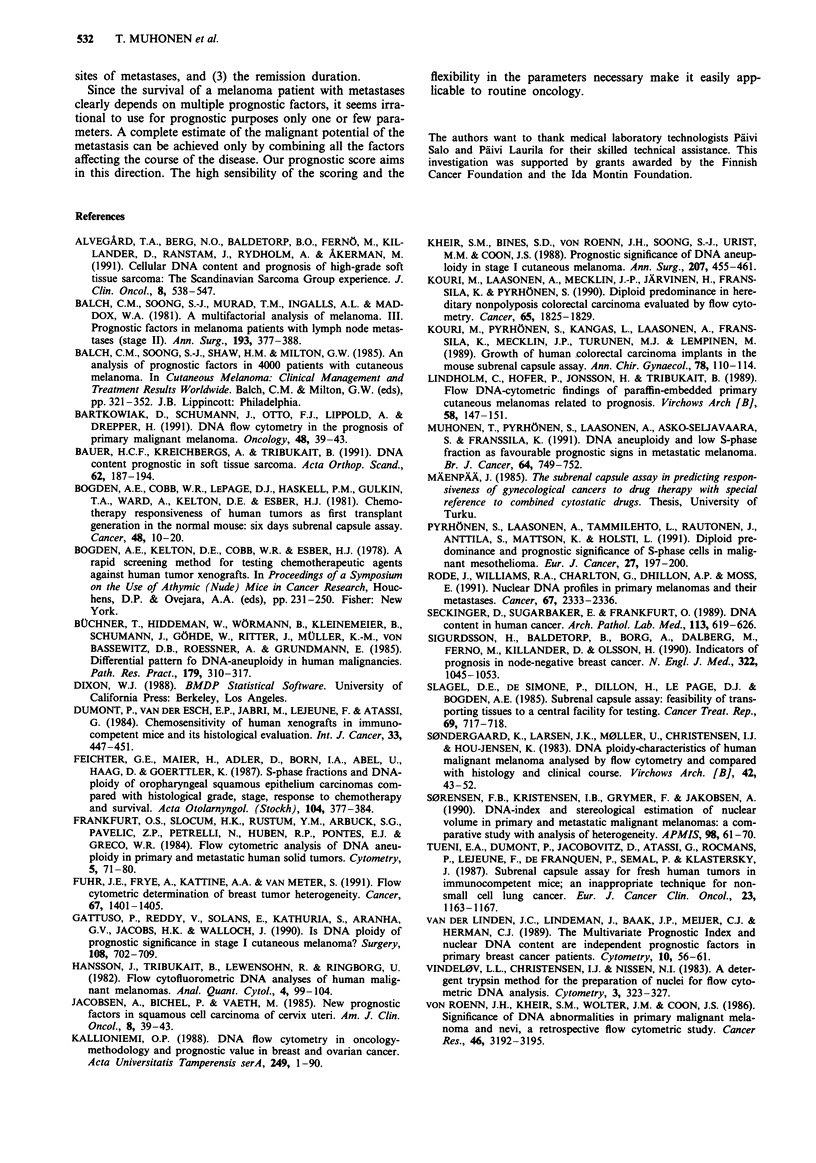# Tumour growth rate and DNA flow cytometry parameters as prognostic factors in metastatic melanoma.

**DOI:** 10.1038/bjc.1992.307

**Published:** 1992-09

**Authors:** T. Muhonen, S. Pyrhönen, A. Laasonen, V. M. Wasenius, S. Asko-Seljavaara, K. Franssila, L. Kangas

**Affiliations:** Department of Radiotherapy and Oncology, Helsinki University Central Hospital, Finland.

## Abstract

The prognostic value of flow cytometric parameters and tumour growth rate of melanoma metastases under the mouse renal capsule was investigated for tumours from 117 consecutive patients referred to the Helsinki University Central Hospital Melanoma Team. DNA flow cytometry (FCM) was interpretable for the tumours of 114 patients, and growth rate analysis for 82 patients, both results being available from 79 patients. Thirty-six percent of the tumours were DNA diploid and 64% DNA aneuploid. Tumour ploidy and S-phase fraction were shown by multivariate Cox model analysis to be independent prognostic variables and major determinants of survival after first recurrence. Patients with DNA diploid or aneuploid tumours survived a median 16 and 27 months, respectively. A high growth rate of tumour sample in vivo under the mouse renal capsule tended to be a sign of poor prognosis, although not reaching statistical significance. Combining the results of FCM, tumour growth rate and TNM stage, we propose a highly efficient prognostic scoring method. Patients with a score above 0.75 had a median survival of 11 months compared to 30 months among patients scoring under 0.75 (P less than 0.0001). This score was the most significant (P less than 0.0001) prognostic factor in the Cox model when TNM stage, age, ploidy, SPF, and tumour growth rate were analysed as covariates.


Br. J. Cancer (1992), 66, 528-532 © Macmillan Press Ltd., 1992

### Tumour growth rate and DNA flow cytometry parameters as prognostic factors in metastatic melanoma

T. Muhonen[1], S. Pyrhönen[1], A. Laasonen[2], V.-M. Wasenius[2], S. Asko-Seljavaara[3], K. Franssila[2] & L. Kangas[4]

[1]*Department of Radiotherapy and Oncology, Helsinki University Central Hospital, SF-00290 Helsinki, Finland* [2]*Pathology Laboratory of the Department of Radiotherapy and Oncology, Helsinki University Central Hospital, SF-00290 Helsinki;* [3]*Department of Plastic Surgery, Helsinki University Central Hospital, SF-00290 Helsinki,* [4]*Orion Corporation FARMOS R&D Pharmaceuticals, PO Box 425, SF-20101 Turku, Finland*

**Summary** The prognostic value of flow cytometric parameters and tumour growth rate of melanoma metastases under the mouse renal capsule was investigated for tumours from 117 consecutive patients referred to the Helsinki University Central Hospital Melanoma Team. DNA flow cytometry (FCM) was interpretable for the tumours of 114 patients, and growth rate analysis for 82 patients, both results being available from 79 patients. Thirty-six percent of the tumours were DNA diploid and 64% DNA aneuploid. Tumour ploidy and S-phase fraction were shown by multivariate Cox model analysis to be independent prognostic variables and major determinants of survival after first recurrence. Patients with DNA diploid or aneuploid tumours survived a median 16 and 27 months, respectively. A high growth rate of tumour sample *in vivo* under the mouse renal capsule tended to be a sign of poor prognosis, although not reaching statistical significance. Combining the results of FCM, tumour growth rate and TNM stage, we propose a highly efficient prognostic scoring method. Patients with a score above 0.75 had a median survival of 11 months compared to 30 months among patients scoring under 0.75 ($P < 0.0001$). This score was the most significant ($P < 0.0001$) prognostic factor in the Cox model when TNM stage, age, ploidy, SPF, and tumour growth rate were analysed as covariates.

Malignant melanoma is known for its varying clinical course and its resistance to most non-surgical therapeutic approaches. A few parameters, such as the number of metastatic lymph nodes and the sites of metastases, are known to predict the clinical behaviour of advanced melanoma (Balch *et al.*, 1985).

Flow cytometric analysis of nuclear DNA content and S-phase fraction (SPF) is a feasible method of estimating the malignant potential and growth characteristics of variable malignant tumours (Seckinger *et al.*, 1989). In many tumours DNA aneuploidy and high S-phase fraction (SPF) are reported to correlate with poor prognosis. DNA aneuploid primary melanomas recur earlier and more frequently than do DNA diploid melanomas (Büchner *et al.*, 1985; Frankfurt *et al.*, 1984; Gattuso *et al.*, 1990; Kheir *et al.*, 1988; Lindholm *et al.*, 1989; Søndergaard *et al.*, 1983; von Roenn *et al.*, 1986). DNA aneuploidy has also been associated with shorter survival in primary melanoma (Bartkowiak *et al.*, 1991; Lindholm, 1989; Søndergaard *et al.*, 1983). Patients with DNA aneuploid mestastatic melanoma have also had, according to one report, worse prognosis than did patients with DNA diploid tumours (Søndergaard *et al.*, 1983), while another study showed no association of DNA ploidy but only of the S-phase fraction with prognosis (Hansson *et al.*, 1982). In our own study we found that in contrast to earlier reports DNA aneuploidy correlated with favourable prognosis (Muhonen *et al.*, 1991).

The mouse subrenal capsule assay (SRCA) originally described by Bogden *et al.* (1981; 1978) has been used as an experimental tool to screen efficacy of antineoplastic drugs. However, change in tumour size under the renal capsule of saline-treated mice can also give an estimate of the growth rate and thus of the aggressiveness of the tumour.

We have correlated the tumour growth rate under the mouse renal capsule as well as flow-cytometrically-determined DNA ploidy and S-phase fraction with the clinical outcome of patients with metastatic melanoma.

### Materials and methods

### *Patients*

The group studied consisted of 117 consecutive patients (68 male, 49 female) with metastatic melanoma treated and subsequently followed up at the Department of Plastic Surgery and at the Department of Radiotherapy and Oncology of Helsinki University Central Hospital between August 1983 and June 1991. Tumour specimens were obtained when regional or distant metastases were found. The major characteristics of the patients are presented in Table I.

### *Tumour samples*

Fresh tumour samples from 117 patients were analysed. The specimens for growth rate testing were cut into 3-4 mm

**Table I** Patient characteristics

| *Total no. of patients* | *117* |
|---|---|
| No. of patients with SRCA | 82 |
| No. of patients with FCM | 114 |
| No. of patients with both SRCA and FCM available | 79 |
| Male/female | 68/49 |
| Mean age (range) | 54 (25-84) |
| Stage at recurrence[a] | |
| III, lymph node involvement | 58 |
| IV | 59 |
| Status at end of follow up (median follow up time) | |
| Alive | 38 (31 months) |
| Deceased | 79 (15 months) |
| Sites of biopsies | |
| Lymph nodes | 72 |
| Cutaneous or subcutaneous | 33 |
| Lung | 3 |
| Other | 9 |

[a]Including eight patients with metastatic disease at the primary admission to clinic.

Correspondence: T. Muhonen, Department of Radiotherapy and Oncology, Helsinki University Central Hospital, Haartmaninkatu 4, SF-00290 Helsinki, Finland
Received 13 January 1992; and in revised form 20 April 1992.

pieces and transferred to a cell-culture medium for transport at a room temperature to the Farmos cancer research laboratory, Turku, Finland. Medium 199 (KC Biological, Lenexa, Ks, USA) supplemented with antibiotics (penicillin + streptomycin or gentamycin) was used in all phases of tumour handling. Implantation of the tumour was accomplished within 24 h. This procedure was adopted on the basis of the experience of the Farmos laboratory (Mäenpää, 1985) as well as that of others (Slagel *et al.*, 1985). A total of 3708 implants of 103 tumours obtained from 82 melanoma patients were analysed.

The samples for flow cytometric DNA analysis were immediately frozen in liquid nitrogen and stored thereafter at −80°C until analysed. DNA analysis was interpretable for 72 individuals of the whole growth-rate tested group. All the specimens were confirmed as representing melanoma by examining the adjacent sections histologically.

*Tumour growth rate under mouse renal capsule*

The method of implanting human tumours under the kidney capsule of mice has been described by Bogden *et al.* (1981). Normal immunocompetent BDF1 and CDF1 hybrid mice provided by the Laboratory Animal Center, Kuopio, Finland, or Alab, Sollentuna, Sweden, and weighing 20–30 g, were used as recipients. Briefly, the mice were anaesthetised with chloral hydrate (370 mg $kg^{-1}$ i.p.), the left kidney was partially exteriorised and one or two 1 $mm^3$ tumour fragments were implanted under the capsule. The size of the implant was determined *in situ* by a stereomicroscope fitted with an ocular micrometer. The change in tumour size (Δts) as ocular microscopic units (omu) was calculated as the difference between the final and initial tumour sizes. The initial and final body weight were measured to evaluate toxicity. For 13 patients, more than one tumour sample was analysed. Only the results of the primary assay are used in this analysis. The assays were carried out in four separate subsequent series. Since each of the series showed a different level of change in tumour size (Δts), a straightforward comparison of the results was not possible. Thus a new parameter: relative growth rate was calculated:

$$\text{Relative growth rate} = \frac{\text{observed individual change in tumour size } (\Delta ts)}{\text{median change in tumour size } (\Delta ts) \text{ of the series}} * 100\%$$

*Flow cytometric analysis*

For analysis the tumour samples were rapidly thawed in a 37°C water bath and processed immediately by scalpel and scissors into single cell suspensions. Each cell suspension was filtered through a 50 μm nylon mesh. The filtrate was centrifuged for 5 min at 1600 r.p.m. Chicken and trout red blood cells were added as internal standards (Vindeløv *et al.*, 1983).

The pellet was resuspended by adding 0.5 ml of ethidium bromide (50 μg $ml^{-1}$ in 10 mM Tris buffer, 1 mM EDTA, 0.3% Nonident P40, pH 7.5). The tube was vortexed and held on ice for 15 min. Then 0.25 ml of a solution containing 1 mg $ml^{-1}$ RNAse (Sigma) was added to the tube, which was incubated for 15 min at room temperature. Immediately before analysis the sample was filtered through a 30 μm nylon mesh. Ten samples were prepared from paraffin-embedded material according to the procedure described previously (Kouri *et al.*, 1990). Routinely at least 15,000 cells per sample were analysed using an EPICS C flow cytometer (Coulter, Hialeah, Florida). A 2 W argon ion laser was used for excitation at a wavelength of 488 nm, and the total emission above 590 nm was measured.

The flow cytometric parameters evaluated included the DNA ploidy and DNA index (DI, where DI represents the ratio of the aneuploid stem line G1 DNA peak channel to the diploid stem line G1-DNA peak channel). Tumours were classified as aneuploid if there occurred a second G1-peak in addition to the diploid G1-peak. Aneuploid tumours with a DI between 1.9 and 2.1 and a definable S- and G2M-phase were defined as tetraploid. The S-phase fraction (SPF) was calculated as described previously (Pyrhönen *et al.*, 1991). When multiple samples per patient were available the average SPF value was used, and the DNA ploidy of the patient was coded as the most deviant DNA ploidy among those samples. Samples with a coefficient of variation greater than 8.0% or with a large amount of debris or with near diploid aneuploidy were excluded from the cell cycle analysis. Since the distribution of SPF values differed significantly between DNA diploid and aneuploid tumours, a new parameter, SPF index, was calculated, dividing the SPF values into two groups based on whether the individual patients' SPF was over or under the median SPF of his DNA ploidy group.

*Statistical methods*

Differences between mean values were analysed using Student's *t*-test, and differences between frequencies using the method of contingency tables.

For calculation of survival after metastases, product-limit survival analysis was performed using the BMDP IL computer program (Dixon, 1988). Brookmeyer-Crawley 95% confidence limits for median survival time are reported. Calculations of the significance of observed differences were performed using the log rank test (Mantel-Cox). *P*-values under 0.05 were considered statistically significant. Cox multivariate analysis was performed with the BMDP 2L computer program using DNA ploidy, SPF index, relative growth rate under the renal capsule, TNM stage, sex, age, disease-free survival and tumour pigmentation as covariates.

### Results

*Tumour growth rate under mouse renal capsule and DNA flow cytometry*

The median change in tumour size, Δts (range) was 1.1 omu (−0.9–8.3) in the first series ($n = 20$), 1.6 omu (0.3–10.2) in the second series ($n = 38$), 2.9 omu (1.0–8.3) in the third series ($n = 16$) and 7.2 omu (2.5–13.8) in the final series ($n = 7$).

A single diploid clone was detected in 41 patients, while 50 other patients exhibited a single aneuploid clone in addition to the diploid one. A further eight patients had a tetraploid clone, and 15 showed multiple aneuploid clones.

The S-phase fraction was calculable for 91 patients. The DNA diploid tumours ($n = 39$) showed a significantly ($P =$ 0.0001) lower SPF than did DNA aneuploid ones ($n = 52$), median 10.1 and 16.8 respectively.

The median growth rates of DNA diploid and DNA aneuploid tumours were 2.0 omu (−0.05–13.8) and 2.2 (−0.9–10.2), respectively. The difference is not statistically significant, nor was correlation detected between SPF value and growth rates.

*Tumour growth rate under mouse renal capsule and survival after metastases*

Patients with rapidly growing tumour specimens tended to have less favourable prognosis. Median survival after metastases of patients whose tumour growth rate exceeded the median of its series was 22 months (95% confidence 15–35) compared to 31 months (95% confidence 25–39) among patients with slowly growing tumours (Figure 1). This difference does not, however, reach the level of significance ($P > 0.05$, log-rank test).

*FCM results and survival after metastases*

DNA aneuploidy was associated with favourable prognosis. Median survival of patients with DNA diploid metastases was 16 months (95% confidence 13–25) compared to 27 months (95% confidence 23–36) for patients with aneuploid metastases; the difference is not, however, significant ($P >$

**Figure 1** Cumulative proportion of melanoma patients surviving after the appearance of first metastases. **a**, Patients with tumours growing slowly under the mouse renal capsule (-■-, $n = 41$) having better survival than those with rapidly growing tumours (-O-, $n = 41$) ($P > 0.05$, product limit, Mantel-Cox). **b**, Patients with DNA aneuploid tumours (-■-, $n = 73$) having better survival than those with DNA diploid tumours (-O-, $n = 41$) ($P > 0.05$, product limit, Mantel-Cox). **c**, Patients with SPF below or at the median (-■-, $n = 46$) showing significantly better prognosis than patients with SPF above median (-O-, $n = 45$) ($P < 0.002$, product limit, Mantel-Cox). **d**, TNM stage III patients (-■-, $n = 58$) showing significantly better prognosis than patients with stage IV disease (-O-, $n = 59$) ($P < 0.01$, product limit, Mantel-Cox, $P > 0.05$ in multivariate analysis with SPF index, ploidy and relative growth rate as covariates). **e**, Patients with prognostic four-variable score at or above 0.75 (-▲-, $n = 26$) having significantly less favourable prognosis than patients scoring between 0.25 and 0.75 (-O-, $n = 80$) as well as patients scoring below 0.25 (-■-, $n = 15$). ($P < 0.0001$ product limit, Mantel-Cox). **f**, Patients with prognostic three-variable score at or above 0.75 (-▲-, $n = 12$) having significantly less favourable prognosis than patients scoring between 0.25 and 0.75 (-O-, $n = 80$) as well as patients scoring below 0.25 (-■-, $n = 25$). ($P < 0.0001$ product limit, Mantel-Cox).

0.05 log rank) (Figure 1). The S-phase fraction (SPF) alone was not a significant prognostic factor, but when the imbalance between different DNA ploidies' SPF was controlled for by adjusting the individual SPF with respect to the median SPF of each respective DNA ploidy group SPF index was also a highly significant prognostic factor. Patients with SPF above the median had a median survival of only 16 months (95% confidence 12–25) compared with 36 months (95% confidence 21–43) among low-SPF patients. The difference is highly significant ($P < 0.002$, log rank test) (Figure 1).

*Multivariate analysis*

Age, TNM stage, DNA ploidy, SPF index and relative growth rate were entered as covariates in Cox's multivariate analysis. SPF above the median was the most important determinant of poor prognosis ($P < 0.002$), followed by DNA diploidy ($P < 0.05$) and TNM stage IV ($P > 0.05$) (Figure 1), and high relative growth rate under mouse renal capsule ($P > 0.05$).

*Prognostic scoring system*

We developed a prognostic scoring system combining the results of DNA flow cytometry and tumour growth rate with the clinical TNM stage of the tumour. A patient scored 1 point for each of the following features: SPF above respective ploidy group median, TNM stage IV, DNA diploidy, and relative growth rate above the median. The crude score obtained is then divided by the number of parameters available for that individual patient. The resulting score ranges from 0 to 1. In a patient scoring 0 all the observed parameters indicate favourable prognosis, and in a patient scoring 1 all the parameters indicate unfavourable prognosis.

The score is a highly significant prognostic tool in the Cox model ($P < 0.0001$). Patients scoring at or above 0.75 have a median survival of 11 months (95% confidence 6–13) compared with 29 months (95% confidence 16-?) among patients scoring below 0.25 (Figure 1).

If the data on growth rate are omitted, and the scoring is based solely on DNA flow cytometry parameters and clinical TNM stage, the scoring separates subpopulations with an even more different prognosis (Table II, Figure 1).

### Discussion

In the present study we analysed 117 melanoma patients' metastases with DNA flow cytometry and determined the growth rate of the tumours *in vivo* under the mouse renal capsule. The observations were correlated with the clinical outcome of these patients. We also propose an efficient scoring method for determining the prognosis of metastatic melanoma patients.

In our previous study on basically the same patient population we observed that DNA aneuploid metastatic melanoma patients exhibited a more favourable prognosis than did DNA diploid patients (Muhonen *et al.*, 1991). This observation is in contrast to most of the studies on primary melanoma, where diploidy is associated with better prognosis (Bartkowiak *et al.*, 1991; Büchner *et al.*, 1985; Frankfurt *et al.*, 1984; Kheir *et al.*, 1988; Lindholm *et al.*, 1989; Søndergaard *et al.*, 1983; von Roenn *et al.*, 1986). Significance of aneuploidy as an independent adverse prognostic sign has,

**Table II** Relationship of DNA flow cytometric and clinical parameters to survival after first melanoma metastases using Brookmeyer-Crawley 95% confidence limits for median survival and product limit Mantel-Cox test for survival analysis

| | *n* | *Median survival* | *95% confidence* | *Observed/ expected* | $\chi^2$ | *P* |
|---|---|---|---|---|---|---|
| DNA ploidy | | | | | | |
| Diploid | 41 | 16 | 13–25 | 1.29 | | |
| Aneuploid | 73 | 27 | 23–36 | 0.88 | 2.65 | >0.05 |
| SPF index | | | | | | |
| Above median | 45 | 16 | 12–25 | 1.53 | | |
| At or below median | 46 | 36 | 22–43 | 0.70 | 10.4 | 0.0013 |
| Tumour growth rate | | | | | | |
| Above median | 41 | 22 | 15–35 | 1.16 | | |
| At or below median | 41 | 31 | 25–39 | 0.87 | 1.3 | >0.05 |
| Stage at relapse | | | | | | |
| III | 58 | 27 | 25–37 | 0.73 | | |
| IV | 59 | 13 | 11–27 | 1.38 | 8.35 | 0.0038 |
| Sex | | | | | | |
| Female | 49 | 25 | 15–35 | 0.98 | | |
| Male | 68 | 25 | 16–31 | 1.01 | 0.01 | >0.05 |
| Score (3 parameters) | | | | | | |
| <0.25 | 25 | 35 | 23- | 0.63 | | |
| 0.25–0.75 | 80 | 26 | 18–31 | 1.02 | | |
| ⩾0.75 | 12 | 6 | 5–11 | 4.46 | 32.02 | <0.0001 |
| Score (4 parameters) | | | | | | |
| <0.25 | 15 | 29 | 16- | 0.59 | | |
| 0.25–0.75 | 76 | 31 | 25–37 | 0.86 | | |
| ⩾0.75 | 26 | 11 | 6–13 | 2.43 | 23.37 | <0.0001 |

however, been challenged in the latest reports (Gattuso *et al.*, 1990; Rode *et al.*, 1991). In contrast to the present study of metastases, all but one (Søndergaard *et al.*, 1983) of the studies reporting poor prognosis of aneuploid tumours have been done on primary lesions. Since the primary operation as well as the histopathological diagnosis was performed in variable institutions, these samples were not accessible for the present analysis.

Like the DNA ploidy pattern, SPF has been described to have prognostic value for metastatic melanomas (Hansson *et al.*, 1982; Muhonen *et al.*, 1991). In the present study, low SPF was observed to be an indicator of a good prognosis. This was seen only after stratification by ploidy. DNA diploid tumours had in general lower SPF values, but less favourable prognosis than did DNA aneuploid tumours. However, within each ploidy group patients with lower SPF had significantly more favourable prognosis.

The clinical relevance of subrenal capsule assay is controversial. In studies on non-small cell lung carcinomas (Tueni *et al.*, 1987) and on colorectal carcinoma (Kouri *et al.*, 1989) a high proportion of the tumour specimens contained no viable tumour cells after the 6 day assay despite the fact that they were optically measured to have grown under the renal capsule during the assay. In contrast, in the study by Dumont and coworkers all the melanoma specimens in the control group were confirmed to contain tumour cells at the end of the 6-day assay (Dumont *et al.*, 1984).

Another source of uncertainty is that the DNA ploidy of samples taken from different areas of a tumour varies considerably (Fuhr *et al.*, 1991; Kallioniemi, 1988; Sørensen *et al.*, 1990). Thus the piece of tumour placed under the renal capsule may actually represent only a diploid clone, although a larger specimen of the same tumour analysed by DNA flow cytometry also shows an aneuploid clone in addition to the diploid clone.

In addition, the S-phase fraction varies considerably in different samples of the same tumour (Kallioniemi, 1988). This could in part explain the observed lack of correlation between the SPF and growth rate. One has to bear in mind that SPF does not directly indicate the growth rate of the tumour, but merely indicates the proportion of cells synthesising DNA. Thus the potential doubling based on BRDU labelling might correlate more clearly with growth rate. Unfortunately BRDU labelling results are available for only a minority of our patients.

Mouse subrenal capsule assay has been generally used for determining the chemosensitivity of a tumour, but not for determining the prognosis of the patient (Bogden *et al.*, 1978; Mäenpää, 1985). Despite this, it is theoretically reasonable to expect that tumours that grow quickly under the mouse kidney capsule would also grow aggressively *in vivo*. Not surprisingly, in our patient population a markedly high growth rate was clearly associated with unfavourable prognosis.

The reason for the increase in growth rate in consecutive series remains unclear. No conscious modifications in the methodology have been performed. The handling of the tumour specimen may, however, have improved over the years, resulting in more nearly optimal growth conditions of the tumour specimen.

As a result of multivariate analysis we propose a simple but effective prognostic scoring method, which combines the results of DNA flow cytometry and tumour growth rate with TNM stage. Because all the parameters are not necessary for scoring, patients for whom growth rate data is missing for example can be scored and compared with cases with complete data. DNA flow cytometry is today available in most pathological units as a routine method, but for routine clinical use the scoring might be more easily applicable when the labour consuming *in vivo* growth rate analysis is omitted. Similar approaches combining FCM and other prognostic parameters have been reported in oropharyngeal squamous epithelium carcinomas (Feichter *et al.*, 1987), soft tissue sarcomas (Alvegård *et al.*, 1991; Bauer *et al.*, 1991), squamous cell carcinoma of cervix uteri (Jacobsen *et al.*, 1985) and breast carcinoma (Sigurdsson *et al.*, 1990; van der Linden *et al.*, 1989) but not in melanoma. Indeed, there are few parameters with any prognostic value in metastatic melanoma. In the large studies by Balch and colleagues (Balch *et al.*, 1981; Balch *et al.*, 1985) the only parameters that significantly predicted the survival after the appearance of lymph node metastases were (1) number of affected nodes (2) ulceration of the primary lesion, and after the appearance of distant metastases were (1) the number of metastatic sites, (2) the sites of metastases, and (3) the remission duration.

Since the survival of a melanoma patient with metastases clearly depends on multiple prognostic factors, it seems irrational to use for prognostic purposes only one or few parameters. A complete estimate of the malignant potential of the metastasis can be achieved only by combining all the factors affecting the course of the disease. Our prognostic score aims in this direction. The high sensibility of the scoring and the flexibility in the parameters necessary make it easily applicable to routine oncology.

The authors want to thank medical laboratory technologists Päivi Salo and Päivi Laurila for their skilled technical assistance. This investigation was supported by grants awarded by the Finnish Cancer Foundation and the Ida Montin Foundation.

### References

ALVEGÅRD, T.A., BERG, N.O., BALDETORP, B.O., FERNÖ, M., KILLANDER, D., RANSTAM, J., RYDHOLM, A. & ÅKERMAN, M. (1991). Cellular DNA content and prognosis of high-grade soft tissue sarcoma: The Scandinavian Sarcoma Group experience. *J. Clin. Oncol.*, **8**, 538–547.

BALCH, C.M., SOONG, S.-J., MURAD, T.M., INGALLS, A.L. & MADDOX, W.A. (1981). A multifactorial analysis of melanoma. III. Prognostic factors in melanoma patients with lymph node metastases (stage II). *Ann. Surg.*, **193**, 377–388.

BALCH, C.M., SOONG, S.-J., SHAW, H.M. & MILTON, G.W. (1985). An analysis of prognostic factors in 4000 patients with cutaneous melanoma. In *Cutaneous Melanoma: Clinical Management and Treatment Results Worldwide*. Balch, C.M. & Milton, G.W. (eds), pp. 321–352. J.B. Lippincott: Philadelphia.

BARTKOWIAK, D., SCHUMANN, J., OTTO, F.J., LIPPOLD, A. & DREPPER, H. (1991). DNA flow cytometry in the prognosis of primary malignant melanoma. *Oncology*, **48**, 39–43.

BAUER, H.C.F., KREICHBERGS, A. & TRIBUKAIT, B. (1991). DNA content prognostic in soft tissue sarcoma. *Acta Orthop. Scand.*, **62**, 187–194.

BOGDEN, A.E., COBB, W.R., LEPAGE, D.J., HASKELL, P.M., GULKIN, T.A., WARD, A., KELTON, D.E. & ESBER, H.J. (1981). Chemotherapy responsiveness of human tumors as first transplant generation in the normal mouse: six days subrenal capsule assay. *Cancer*, **48**, 10–20.

BOGDEN, A.E., KELTON, D.E., COBB, W.R. & ESBER, H.J. (1978). A rapid screening method for testing chemotherapeutic agents against human tumor xenografts. In *Proceedings of a Symposium on the Use of Athymic (Nude) Mice in Cancer Research*, Houchens, D.P. & Ovejara, A.A. (eds), pp. 231–250. Fisher: New York.

BÜCHNER, T., HIDDEMAN, W., WÖRMANN, B., KLEINEMEIER, B., SCHUMANN, J., GÖHDE, W., RITTER, J., MÜLLER, K.-M., VON BASSEWITZ, D.B., ROESSNER, A. & GRUNDMANN, E. (1985). Differential pattern fo DNA-aneuploidy in human malignancies. *Path. Res. Pract.*, **179**, 310–317.

DIXON, W.J. (1988). *BMDP Statistical Software*. University of California Press: Berkeley, Los Angeles.

DUMONT, P., VAN DER ESCH, E.P., JABRI, M., LEJEUNE, F. & ATASSI, G. (1984). Chemosensitivity of human xenografts in immunocompetent mice and its histological evaluation. *Int. J. Cancer*, **33**, 447–451.

FEICHTER, G.E., MAIER, H., ADLER, D., BORN, I.A., ABEL, U., HAAG, D. & GOERTTLER, K. (1987). S-phase fractions and DNA-ploidy of oropharyngeal squamous epithelium carcinomas compared with histological grade, stage, response to chemotherapy and survival. *Acta Otolarnyngol. (Stockh)*, **104**, 377–384.

FRANKFURT, O.S., SLOCUM, H.K., RUSTUM, Y.M., ARBUCK, S.G., PAVELIC, Z.P., PETRELLI, N., HUBEN, R.P., PONTES, E.J. & GRECO, W.R. (1984). Flow cytometric analysis of DNA aneuploidy in primary and metastatic human solid tumors. *Cytometry*, **5**, 71–80.

FUHR, J.E., FRYE, A., KATTINE, A.A. & VAN METER, S. (1991). Flow cytometric determination of breast tumor heterogeneity. *Cancer*, **67**, 1401–1405.

GATTUSO, P., REDDY, V., SOLANS, E., KATHURIA, S., ARANHA, G.V., JACOBS, H.K. & WALLOCH, J. (1990). Is DNA ploidy of prognostic significance in stage I cutaneous melanoma? *Surgery*, **108**, 702–709.

HANSSON, J., TRIBUKAIT, B., LEWENSOHN, R. & RINGBORG, U. (1982). Flow cytofluorometric DNA analyses of human malignant melanomas. *Anal. Quant. Cytol.*, **4**, 99–104.

JACOBSEN, A., BICHEL, P. & VAETH, M. (1985). New prognostic factors in squamous cell carcinoma of cervix uteri. *Am. J. Clin. Oncol.*, **8**, 39–43.

KALLIONIEMI, O.P. (1988). DNA flow cytometry in oncology-methodology and prognostic value in breast and ovarian cancer. *Acta Universitatis Tamperensis serA*, **249**, 1–90.

KHEIR, S.M., BINES, S.D., VON ROENN, J.H., SOONG, S.-J., URIST, M.M. & COON, J.S. (1988). Prognostic significance of DNA aneuploidy in stage I cutaneous melanoma. *Ann. Surg.*, **207**, 455–461.

KOURI, M., LAASONEN, A., MECKLIN, J.-P., JÄRVINEN, H., FRANSSILA, K. & PYRHÖNEN, S. (1990). Diploid predominance in hereditary nonpolyposis colorectal carcinoma evaluated by flow cytometry. *Cancer*, **65**, 1825–1829.

KOURI, M., PYRHÖNEN, S., KANGAS, L., LAASONEN, A., FRANSSILA, K., MECKLIN, J.P., TURUNEN, M.J. & LEMPINEN, M. (1989). Growth of human colorectal carcinoma implants in the mouse subrenal capsule assay. *Ann. Chir. Gynaecol.*, **78**, 110–114.

LINDHOLM, C., HOFER, P., JONSSON, H. & TRIBUKAIT, B. (1989). Flow DNA-cytometric findings of paraffin-embedded primary cutaneous melanomas related to prognosis. *Virchows Arch [B]*, **58**, 147–151.

MUHONEN, T., PYRHÖNEN, S., LAASONEN, A., ASKO-SELJAVAARA, S. & FRANSSILA, K. (1991). DNA aneuploidy and low S-phase fraction as favourable prognostic signs in metastatic melanoma. *Br. J. Cancer*, **64**, 749–752.

MÄENPÄÄ, J. (1985). *The subrenal capsule assay in predicting responsiveness of gynecological cancers to drug therapy with special reference to combined cytostatic drugs*. Thesis, University of Turku.

PYRHÖNEN, S., LAASONEN, A., TAMMILEHTO, L., RAUTONEN, J., ANTTILA, S., MATTSON, K. & HOLSTI, L. (1991). Diploid predominance and prognostic significance of S-phase cells in malignant mesothelioma. *Eur. J. Cancer*, **27**, 197–200.

RODE, J., WILLIAMS, R.A., CHARLTON, G., DHILLON, A.P. & MOSS, E. (1991). Nuclear DNA profiles in primary melanomas and their metastases. *Cancer*, **67**, 2333–2336.

SECKINGER, D., SUGARBAKER, E. & FRANKFURT, O. (1989). DNA content in human cancer. *Arch. Pathol. Lab. Med.*, **113**, 619–626.

SIGURDSSON, H., BALDETORP, B., BORG, A., DALBERG, M., FERNO, M., KILLANDER, D. & OLSSON, H. (1990). Indicators of prognosis in node-negative breast cancer. *N. Engl. J. Med.*, **322**, 1045–1053.

SLAGEL, D.E., DE SIMONE, P., DILLON, H., LE PAGE, D.J. & BOGDEN, A.E. (1985). Subrenal capsule assay: feasibility of transporting tissues to a central facility for testing. *Cancer Treat. Rep.*, **69**, 717–718.

SØNDERGAARD, K., LARSEN, J.K., MØLLER, U., CHRISTENSEN, I.J. & HOU-JENSEN, K. (1983). DNA ploidy-characteristics of human malignant melanoma analysed by flow cytometry and compared with histology and clinical course. *Virchows Arch. [B]*, **42**, 43–52.

SØRENSEN, F.B., KRISTENSEN, I.B., GRYMER, F. & JAKOBSEN, A. (1990). DNA-index and stereological estimation of nuclear volume in primary and metastatic malignant melanomas: a comparative study with analysis of heterogeneity. *APMIS*, **98**, 61–70.

TUENI, E.A., DUMONT, P., JACOBOVITZ, D., ATASSI, G., ROCMANS, P., LEJEUNE, F., DE FRANQUEN, P., SEMAL, P. & KLASTERSKY, J. (1987). Subrenal capsule assay for fresh human tumors in immunocompetent mice; an inappropriate technique for non-small cell lung cancer. *Eur. J. Cancer Clin. Oncol.*, **23**, 1163–1167.

VAN DER LINDEN, J.C., LINDEMAN, J., BAAK, J.P., MEIJER, C.J. & HERMAN, C.J. (1989). The Multivariate Prognostic Index and nuclear DNA content are independent prognostic factors in primary breast cancer patients. *Cytometry*, **10**, 56–61.

VINDELØV, L.L., CHRISTENSEN, I.J. & NISSEN, N.I. (1983). A detergent trypsin method for the preparation of nuclei for flow cytometric DNA analysis. *Cytometry*, **3**, 323–327.

VON ROENN, J.H., KHEIR, S.M., WOLTER, J.M. & COON, J.S. (1986). Significance of DNA abnormalities in primary malignant melanoma and nevi, a retrospective flow cytometric study. *Cancer Res.*, **46**, 3192–3195.